# Case Report: Grade 2 Metastatic Pancreatic Neuroendocrine Tumor With Progression of One Metastasis After Pregnancy to Grade 3 Large-Cell Neuroendocrine Carcinoma: One Case Cured by Resection With Genomic Characterization of the Two Components

**DOI:** 10.3389/fonc.2021.646992

**Published:** 2021-03-31

**Authors:** Jean-Luc Raoul, Marie-Françoise Heymann, Frédéric Dumont, Alain Morel, Hélène Senellart, François Bertucci

**Affiliations:** ^1^ Department of Medical Oncology, Institut de Cancérologie de l’Ouest, Saint-Herblain, France; ^2^ Department of Pathology, Institut de Cancérologie de l’Ouest, Saint-Herblain, France; ^3^ Department of Surgery, Institut de Cancérologie de l’Ouest, Saint-Herblain, France; ^4^ Department of Oncopharmacology, Institut de Cancérologie de l’Ouest, Angers, France; ^5^ Predictive Oncology Laboratory, Department of Medical Oncology, CRCM, Institut Paoli-Calmettes, Aix-Marseille Université, Marseille, France

**Keywords:** ****sporadic gastrinoma, metastases, surgery, genomics, MEN 1 gene, microsatellite instability, MLH1

## Abstract

Temporal and spatial tumor heterogeneity can be observed in pancreatic neuroendocrine tumor. We report the case of a young woman with long term stabilization of a G2 metastatic pancreatic NET that, after pregnancy, suddenly progressed into one single liver metastasis corresponding to a transformation into G3 large-cell neuroendocrine cancer. The patient underwent liver resection (the progressive and one dormant metastasis). With a 45 months follow-up the patient is without evolutive disease. Exome sequencing of the two metastases revealed completely different genomic signatures and gene alterations: the dormant metastasis was MSS without any gene alteration; the poorly differentiated tumor was MSI, with gain of many mutations including MEN1, BCL2, MLH1 and TP53 corresponding to a mutational signature 11. Could temozolomide play a role in this transformation?

## Introduction

Gastroenteropancreatic neuroendocrine neoplasia (NEN) are low in incidence but high in prevalence due to their usual good prognosis even when metastatic (1). They represent a very heterogeneous group of tumors, particularly regarding their behavior. The pathological features, mainly based on proliferation index (assessed by Ki67 immunohistochemistry labeling) and differentiation, have a major prognostic value (2). The WHO 2017 grading classification for pancreatic NEN (3) endorses the WHO 2010 principles. Neuroendocrine tumors (NETs) are well differentiated and composed of three grades (G): G1 (Ki67 <3%), G2 (Ki67 3%–20%), and G3 (>20%). Neuroendocrine carcinomas (NEC) correspond to poorly differentiated tumors, by default G3, and can involve large-cell or small-cell types. They differ from NETs with respect to clinical and biological features, outcome, and treatment. Yet some problems remain, including temporal and spatial tumor heterogeneity. The disease can evolve with time from grades G1–G2 to G3 and eventually toward a poorly differentiated NEC. The grade can differ between different sites in the same tumor and the same patient, and heterogeneity is even more frequent between the primary and the metastases, particularly when metachronous (4). A molecular classification will certainly be helpful in the near future (5).

Here, we report the case of a young woman with very long-term stabilization of a G2 metastatic pancreatic NET that, after pregnancy, suddenly progressed into one single liver metastasis corresponding to a transformation into G3 large-cell NEC. The patient underwent liver resection (the progressive and one dormant metastases). Exome sequencing of the two metastases revealed completely different genomic signatures and gene alterations.

## Case Report

Written informed consent for publication of her clinical details and images was obtained from the patient.

A 22-year-old female patient was seen in October 2009 for chronic diarrhea and weight loss (-8 kg). Colonoscopy was normal, but upper endoscopy revealed esophageal and duodenal ulcerations. CT scan disclosed a 4-cm tumor of the pancreatic tail associated with 10 minute (<1 cm) liver metastases. On endoscopic ultrasound, the pancreatic tumor was unique, and tumor cytology found well-differentiated neuroendocrine tumor cells (low Ki67 at 1%). Serum gastrin and chromogranin A (CgA) levels were elevated (2446 UI and 1455 UI respectively); Neuron-Specific Enolase (NSE), glucagon, insulin, and VIP serum levels were normal. Proton-pump inhibitors (PPIs) treatment was initiated. An oncogenic workup did not find any other familial case and the *MEN 1* gene was not mutated. The diagnosis of sporadic metastatic pancreatic gastrinoma was retained. After Multidisciplinary Tumor Board discussion, a spleno-pancreatectomy with lymphadenectomy and radiofrequency ablation of three metastases located in the right liver were performed in December 2009. Pathological analysis found a pancreatic G2 well-differentiated NET of 7 cm with 3/41 metastatic lymph nodes, a Ki67 index of 10%, and a low mitotic rate (4 mitoses/10 high-power fields). Postoperative serum gastrin and CgA levels remained stable (1104 UI and 1164 UI respectively). On somatostatin receptor scintigraphy, five liver metastases were clearly seen, with no other foci. The patient then received, in combination with PPIs, monthly intramuscular injections of 20 mg octreotide long-acting release (LAR) with a rapid fall in serum tumor markers.

**Figure 1:**
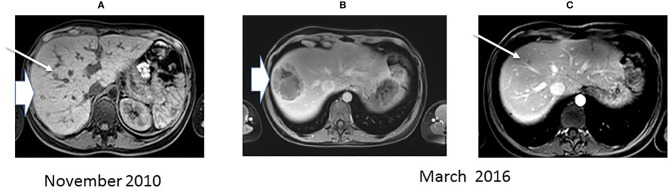
MRI. MRI imaging of the liver in 2010 showing two minute tumors (**A** one progressed (arrowhead) **B**) in March 2016 while the other (thin arrow) **(C)** remained stable.

Three months later, liver magnetic resonance imaging (MRI) found liver progression with the appearance of new lesions. A systemic chemotherapy regimen (6) combining capecitabine (750 mg/m^2^ orally twice a day on days 1–14) and temozolomide (200 mg/m^2^ orally once a day on days 10–14) was given for six 28-day cycles. A total body CT scan and a liver MRI then showed a partial response, and tumor markers returned to normal values. Monthly octreotide LAR was continued with a three-month follow-up liver MRI. On January 2014, the total body CT scan and liver MRI confirmed the partial response (most lesions remained centimetric) (**Figure 1A**), and a ^68^Ga-DOTATOC PET scan did not demonstrate any activity. On January 2015, octreotide LAR was voluntarily stopped because this 28-year-old woman became pregnant.

**Figure 2:**
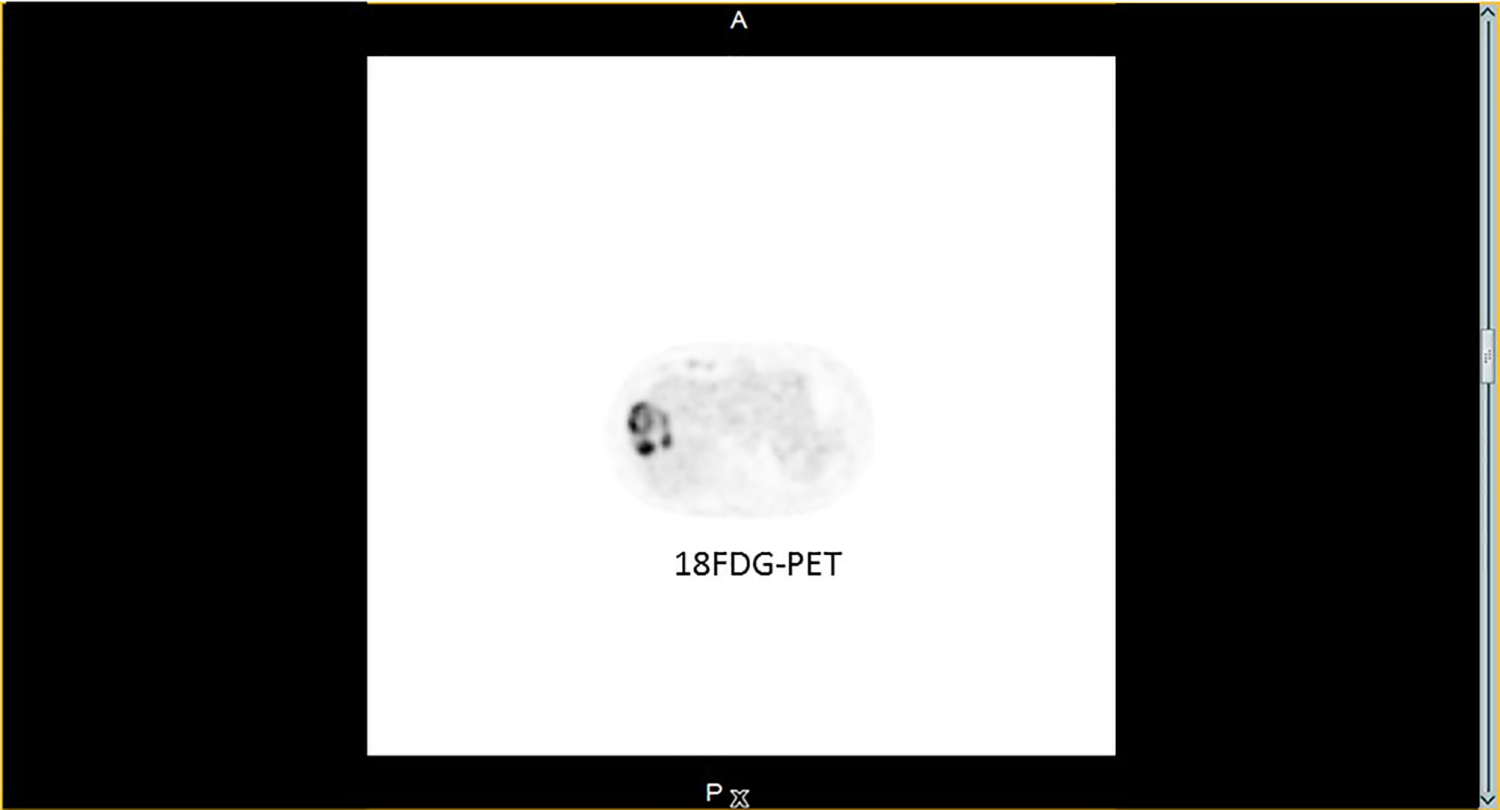
^18^FDG-PET. ^18^FDG-PET image from March 2016: major uptake in the progressing lesion; no other suspect foci was seen.

**Figure 3:**
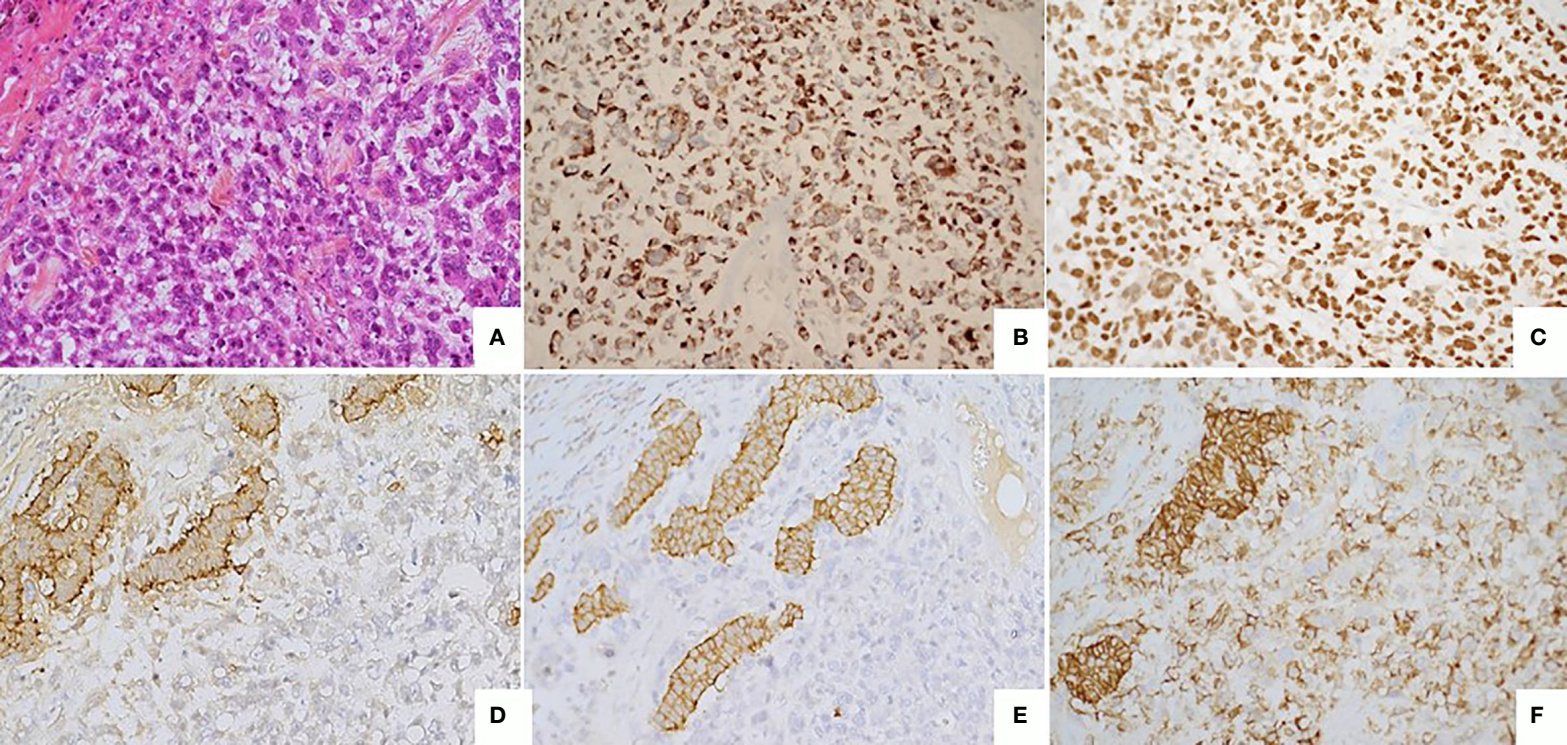
Poorly differentiated tumor **(A)** Large cell neuroendocrine carcinoma composed by large cells with high mitotic index (> 20 mitoses/2mm^2^). HES, x20. **(B)** Immunostaining CKAE1/AE3, monoclonal antibody, clone AE1/AE3/PCK26, Ventana, x20. **(C)** Immunostaining KI67 : high nuclear expression (> 70%) monoclonal antibody, clone MIB1, Agilent Dako, x20. **(D)** Immunostaining Chromogranin A, monoclonal antibody, clone LK2H10, Ventana, X20. **(E)** Immunostaining Synaptophysin, monoclonal antibody, clone SP11, Ventana, X20. **(F)** Immunostaining CD56, monoclonal antibody, clone MRQ-42, Ventana/Cell marque, X20.

**Figure 4:**
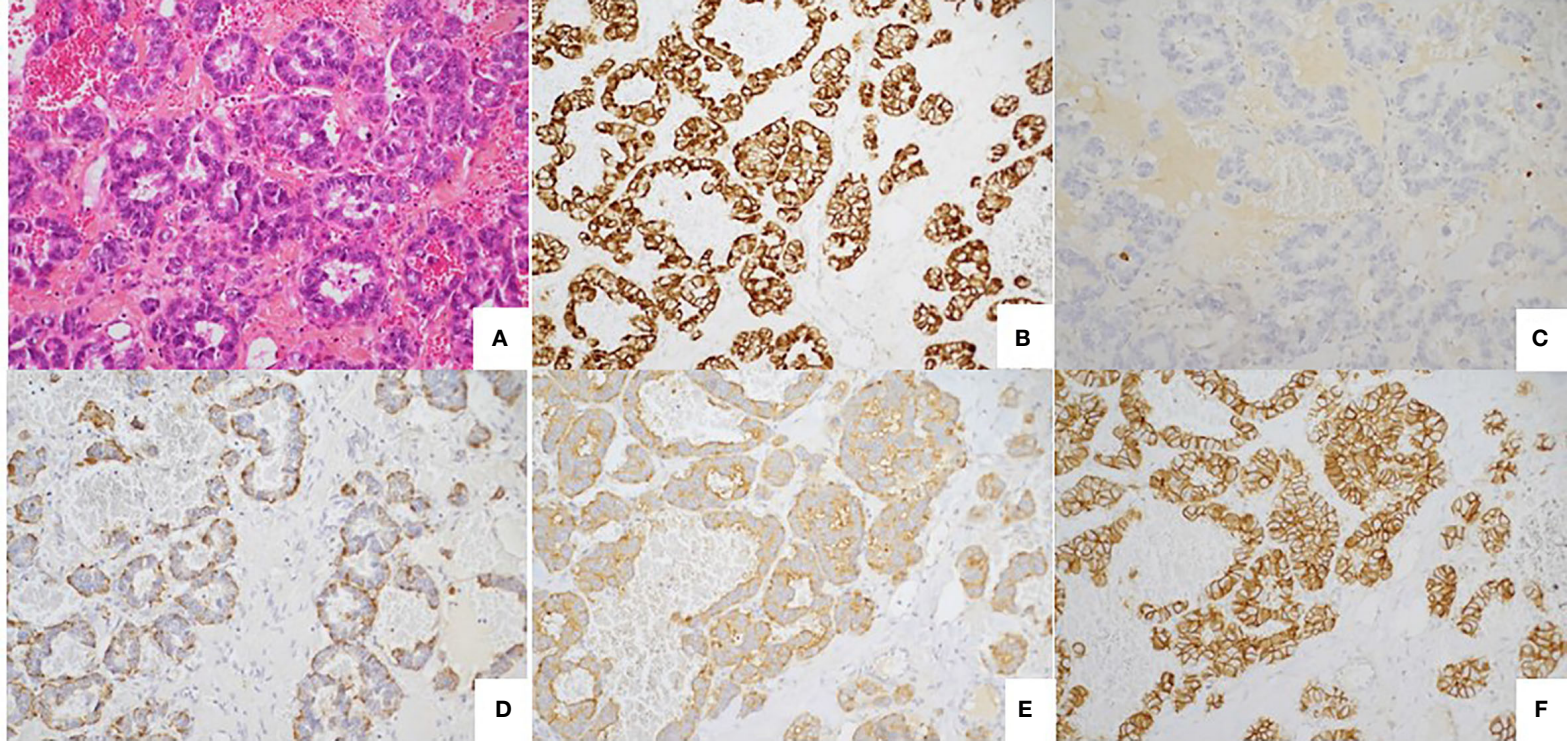
Well differentiated tumor. **(A)** Well-differentiated neuroendocrine tumour with glandular architecture. HES, x 20. **(B)** Immunostaining CKAE1/AE3, monoclonal antibody, clone AE1/AE3/PCK26, Ventana, x20. **(C)** Immunostaining KI67: low nuclear expression (< 1%) monoclonal antibody, clone MIB1, Agilent Dako, x20. **(D)** Immunostaining Chromogranin A, monoclonal antibody, clone LK2H10, Ventana, X20. **(E)** Immunostaining Synaptophysin, monoclonal antibody, clone SP11, Ventana, X20. **(F)** Immunostaining CD56, monoclonal antibody, clone MRQ-42, Ventana/Cell marque, X20.

On December 2015, two months after a normal delivery, an ultrasound scan showed a liver progression with one lesion increasing from 15 mm to 55 mm in diameter. Octreotide LAR was reintroduced, but three months later MRI showed a further increase in the size of the larger lesion with a necrotic appearance (**Figure 1B**), while the other hepatic nodules remained stable (**Figure 1C**). ^18^Fluorodeoxyglucose PET scan showed major hypermetabolism of this nodule (standardized uptake value 22.8) with no activity elsewhere (**Figure 2**). Biopsy was performed on this progressing liver metastasis and showed a well- to moderately differentiated component with a Ki67 index of 14% (G2) surrounding a poorly differentiated NEC with large cells and a Ki67 index of 60%, suggesting a transformation from low-grade NET to high-grade large-cell NEC. The NSE level then began to rise. The combination of capecitabine and temozolomide was reintroduced but failed. After right portal vein embolization, a right hepatectomy was performed in January 2017. The pathological examination identified two tumors separated by more than 20 mm: one was necrotic and measured 75 mm, and the second measured 23 mm. Macroscopically, there were two hepatic tumors, which measured 75 mm and 23 mm in diameter respectively. Both tumors were white colored, with a necrotic consistency for the largest one. The histologic study showed two neuroendocrine proliferations with different differentiations (**Figures 3** and **4**). The larger metastases was poorly differentiated with compact architecture: clumps of tumor cells expressed CD56, TTF-1, p53, and partly Chromogranin A and synaptophysin in some areas. The mitotic index was higher than 20 mitoses per 2 mm^2^ (Ki67 index >80%). The other tumor was well differentiated and characterized by a glandular proliferation expressing the three neuroendocrine markers and by the absence of mitosis. The Ki67 index was low (<1%).

In June 2020, 41 months after resection, the imaging remained stable with no sign of progression.

## Molecular Analysis

We extended the comparison of these two resected metastases at the molecular level by performing exome next-generation sequencing (FoundationOne^®^ CDx, Foundation Medicine) on each lesion. The pathologically and radiologically dormant liver metastasis showed a Microsatellite Stable (MSS) status with a low tumor mutational burden (TMB; three mutations per Mb). No major gene alteration was seen, with only six variants of unknown significance detected on *BRCA2* (K2791N), *FOXL2* (V14I), *KDR* (R1229Q), *KLHL6* (M313I), *MAP3K13* (R458H), and *NOTCH1* (N280S). In contrast, the mutational profile of the poorly differentiated tissue of the progressing metastasis revealed many alterations, including Microsatellite Instable (MSI) status, very high TMB (153 mutations per Mb), and many gene mutations. The later notably involved *MEN1* splice site (927 + 1G>A), *BCL2* (E165K), *BCORL1* (Q606*), *DAXX* splice site (1039 + 1G>A), *ERBB4* (R50H), *HNF1A* (G292fs*25), *KDM5C* (R68fs*5), *MLH1* splice site (207 + 1G>A, Q510*), *MLL2* splice site (5645-1G>A), *PPP2R2A* splice site (180 + 1G>A), and *TP53* (P191del, R280K).

## Discussion

This case demonstrates that, after a long follow-up, a liver metastasis of a G2 functional (gastrinoma) pancreatic NET can dedifferentiate to a poorly differentiated G3 NEC, perhaps stimulated by a pregnancy.

The influence of pregnancy on the evolution of NETs is poorly understood, but these tumors may be sensitive to hormones and proteins produced in excess during the pregnancy. Pancreas NETs frequently express progesterone receptor (PR), and less often estrogen receptor (ER) (7, 8). Recently it was demonstrated (9) that placental growth factor, a member of the VEGF family (10), stimulates the growth of NETs. In the relationship between cancer and pregnancy, many factors are involved, and particularly immune modulations (11). It has also been reported that breast cancers observed during pregnancy frequently have more aggressive clinical behavior, perhaps related to molecular differences (12). Yet the impact of pregnancy on a slow-growing cancer like NET, obviously a rare event, is not described, to the best of our knowledge, in the literature.

If modification from a G1 to G2 NET is not unusual, progression of a G1 or G2 NET to a poorly differentiated NEC has seldom been described. A retrospective series reported 31 cases of well-differentiated digestive tract NETs (at least 50% of the tumor) with a separable component of high-grade NEC (13), including 21 pancreatic NETs. The high-grade part was reported either within the primary tumor (48%) or at metastatic sites (52%), and in most cases constituted at least 20% of the tumor. The high-grade component had no features of small-cell carcinoma in any of these cases but there were sometimes histologic overlaps with large-cell NEC. The prognosis of these patients remained good, closer to that of patients with well-differentiated NETs, and far better than observed in purely high-grade NEC. p53 positivity by IHC, a surrogate for *TP53* mutations, has never been reported in well-differentiated NETs or in these 21 transformed pancreatic NETs, but is found in most poorly differentiated pancreatic NEC and in our case. *DAXX*/*ATRX*/*MEN1* mutations were detected at similar frequency in the high-grade component and the lower-grade equivalent of pancreatic well-differentiated NETs, but never in a series of poorly differentiated pancreatic NEC. The contrary was observed for *RB1* mutations. For the authors, these parts of poorly differentiated NEC are, from a histogenetic point of view, closer to conventional carcinoma (from squamous or glandular cell origin) than to a conventional well-differentiated NET. For others (14), higher-grade regions in epithelial neoplasms reflect neoplastic progression due to additional molecular and genetic events. An alternative explanation is that regional variations in morphology reflect epigenetic variations or multiclonality.

Comparison of the exomes of the two tumor components in our patient gave some important information.

No gene alteration has been described in the well-differentiated part of the tumor. In contrast, the poorly differentiated part was MSI-h with a very high (153 muts/Mb) mutational burden, and many gene mutations, mostly G>A. This tumor was MSI-h, with a MLH1-acquired mutation. Such MLH1-acquired mutations can be found in MSI-h cancers from the Lynch spectrum without germline mutations (or hypermethylations) (15); MLH1 inactivation leads to increased mutational burden resulting in microsatellite instability. Only one case of pancreatic NEC (Ki67 = 60%) with such mismatched repair deficiency and loss of expression of MLH1 and PMS2 in tumor cells without gene promoter methylation has been reported (16); the outcome was surprisingly good. In a series of 89 gastroenteropancreatic NEC or mixed adenoneuroendocrine carcinomas with no familial history of Lynch syndrome, 12.4% had a MSI phenotype (essentially those with a primary located in the stomach or small intestine), usually due to methylation-mediated silencing of the MLH1 gene (17). Their prognosis was good. MLH1 mutation is rare in NEC, reported in less than 1% of small-cell lung carcinomas, in less than 1% of pancreatic NETs, and never in intestinal carcinoids; a few cases of pancreatic NEC with MSI-h in a Lynch syndrome due to germinal MLH1 mutation have been described (18).

MEN1, a tumor-suppressor gene that encodes the protein menin, is frequently mutated in neuroendocrine tumors. Germline mutations are associated with multiple endocrine neoplasia type 1 with frequently pancreatic NETs. Somatic mutations are observed in one third of pancreatic NETs (5) and in only a few cases of pancreatic adenocarcinomas. In our case, at diagnosis, MEN1 germline testing was negative and no alteration of MEN1 was described in the well-differentiated part of the removed tumor. In contrast, MEN1 alteration was found in the second component. The MEN1 splice site 927 + 1G>A has been described on germline allele and considered as likely pathogenic (ClinVar). This loss of menin, an epigenetic regulator, leads to the inactivation of p53/Rb pathways and triggers aberrant DNA damage response (19). DAXX mutations, observed in 20% of pancreatic NETs, are usually correlated with a poor prognosis; they can be driver mutations (5) and play a role in chromosomal instability (5). Other mutations described in our patients are infrequently found in NETs: never for BCL2 mutations, in many other cancers (gastric, prostate cancers, and melanoma) for ERBB4 mutations (coding for a member of the ErbB receptor family), and in endometrial tumors and in liver adenomas for HNF1A mutations.

In our case, most gene mutations were single-base substitution, essentially G>A; this mutational signature (signature 11) is particularly observed in glioblastoma and melanoma resistant to the alkylating agent, temozolomide (20), and our patient received such treatment. More recently, in gliomas with a high tumor burden, two main pathways have led to hypermutation: a *de novo* pathway and more commonly a post-treatment pathway associated with acquired resistance driven by MMR defects after treatment by temozolomide. This signature 11 may be caused, in gliomas, by temozolomide exposure and by MMR deficiency (21), and not by “pure” temozolomide signature. In such hypermutated MMR-deficient glioblastomas, the efficacy of PD-1 blockade seems infrequent (21).

Yet the debate is still open. Lung neuroendocrine carcinoids are being considered as opposed to large-cell NEC or small-cell lung carcinoma. In a recent series, 148 resected lung-neuroendocrine tumors (22), 53 typical carcinoids, 35 atypical carcinoids, 27 large-cell NEC, and 33 small-cell NEC were investigated by NGS. Six histology-independent tumor clusters were found. Based on these results, the authors consider that typical carcinoids have the potential to evolve into high-grade tumors directly or indirectly, perhaps smoking-related. They consider that this phenomenon is inherent to all NETs and so not rare.

Treatment of such local progression is unclear. Local treatment seems appropriate to destroy this aggressive tumor, which differs from other low-grade and stable metastases. A series of 69 patients who received local treatment for focal progression was reported from three NET referral centers (14). The primary was pancreas in 55% of the cases. Most patients had low-grade well-differentiated tumors and none of the resected specimens had a poorly differentiated appearance. Locoregional treatments included tumor ablation (n=19), resection (n=18), embolization (n=16), and external beam radiation (n=16). The site of focal progression was the liver in 75% of the cases. The outcome was good with median progression-free survival of 17 months, and a median time to new systemic therapy of 21 months (for resected patients).

In conclusion, our case confirms that focal progression in metastatic NETs can correspond to a transformation of indolent low-grade tumor into high-grade poorly differentiated tumor, and that local treatment (here surgical resection) can reset the prognosis. Our case also confirms that ^18^Fluoroglucose PET scans (23) can be useful to distinguish these transformed metastases from indolent metastases. Comparison of the exomes of both components shows impressive differences: no major abnormalities in the well-differentiated component, while in the poorly differentiated component the tumor was MSI with a very high tumor burden, somatic mutations in MEN1, MLH1, p53. Could temozolomide played a role in such tumor transformations?

## Patient Perspective

In case of a major progression limited to one metastases in a metastatic well differentiated NET, surgery may be an excellent therapeutic option even if this progressive tumor is poorly differentiated on biopsy. An exome next generation sequencing may be useful; in case of MSI tumor the use of check-point inhibitors blockade is not always of interest.

## Data Availability Statement

The raw data supporting the conclusions of this article will be made available by the authors, without undue reservation.

## Ethics Statement

Ethical review and approval was not required for the study on human participants in accordance with the local legislation and institutional requirements. The patients/participants provided their written informed consent to participate in this study.

## Author Contributions

J-LR, FB, and HS participated in the conception and writing of the study. FB, J-LR, and AM to the interpretation of the data. HS, M-FH, and FD participated in the acquisition of the data. All authors contributed to the article and approved the submitted version.

## Conflict of Interest

The authors declare that the research was conducted in the absence of any commercial or financial relationships that could be construed as a potential conflict of interest.

## References

[1] Lepage C Bouvier AM Phelip JM Hatem C Vernet C Faivre J. Incidence and management of malignant digestive endocrine tumours in a well defined French population. Gut (2004) 53(4):549–53. 10.1136/gut.2003.026401 PMC1774002 15016750

[2] Kidd M Modlin I Oberg K. Towards a new classification of gastroenteropancreatic neuroendocrine neoplasms. Nat Rev Clin Oncol (2016) 13(11):691–705. 10.1038/nrclinonc.2016.85 27273044

[3] Inzani F Petrone G Rindi G. The New World Health Organization Classification for Pancreatic Neuroendocrine Neoplasia. Endocrinol Metab Clin North Am (2018) 47(3):463–70. 10.1016/j.ecl.2018.04.008 30098710

[4] Grillo F Albertelli M Brisigotti MP Borra T Boschetti M Fiocca R. Grade Increases in Gastroenteropancreatic Neuroendocrine Tumor Metastases Compared to the Primary Tumor. Neuroendocrinology (2016) 103(5):452–9. 10.1159/000439434 26337010

[5] Scarpa A Chang DK Nones K Corbo V Patch AM Bailey P. Whole-genome landscape of pancreatic neuroendocrine tumours. Nature (2017) 543(7643):65–71. 10.1038/nature21063 28199314

[6] Ramirez RA Beyer DT Chauhan A Boudreaux JP Wang YZ Woltering EA. The Role of Capecitabine/Temozolomide in Metastatic Neuroendocrine Tumors. Oncologist (2016) 21(6):671–5. 10.1634/theoncologist.2015-0470 PMC4912368 27226359

[7] Zimmermann N Lazar-Karsten P Keck T Billmann F Schmid S Brabant G. Expression Pattern of CDX2, Estrogen and Progesterone Receptors in Primary Gastroenteropancreatic Neuroendocrine Tumors and Metastases. Anticancer Res (2016) 36(3):921–4. 26976979

[8] Estrella JS Ma LT Milton DR Yao JC Wang H Rashid A. Expression of estrogen-induced genes and estrogen receptor beta in pancreatic neuroendocrine tumors: implications for targeted therapy. Pancreas (2014) 43(7):996–1002. 10.1097/MPA.0000000000000203 25058880 PMC4628823

[9] Hilfenhaus G Gohrig A Pape UF Neumann T Jann H Zdunek D. Placental growth factor supports neuroendocrine tumor growth and predicts disease prognosis in patients. Endocr Relat Cancer (2013) 20(3):305–19. 10.1530/ERC-12-0223 23463017

[10] Dewerchin M Carmeliet P. Placental growth factor in cancer. Expert Opin Ther Targets (2014) 18(11):1339–54. 10.1517/14728222.2014.948420 25297943

[11] Holtan SG Creedon DJ Haluska P Markovic SN. Cancer and pregnancy: parallels in growth, invasion, and immune modulation and implications for cancer therapeutic agents. Mayo Clin Proc (2009) 84(11):985–1000. 10.4065/84.11.985 19880689 PMC2770910

[12] Nguyen B Venet D Azim HA Jr Brown D Desmedt C Lambertini M. Breast cancer diagnosed during pregnancy is associated with enrichment of non-silent mutations, mismatch repair deficiency signature and mucin mutations. NPJ Breast Cancer (2018) 4:23. 10.1038/s41523-018-0077-3 30109263 PMC6078984

[13] Tang LH Untch BR Reidy DL O’Reilly E Dhall D Jih L. Well-Differentiated Neuroendocrine Tumors with a Morphologically Apparent High-Grade Component: A Pathway Distinct from Poorly Differentiated Neuroendocrine Carcinomas. Clin Cancer Res (2016) 22(4):1011–7. 10.1158/1078-0432.CCR-15-0548 PMC4988130 26482044

[14] Al-Toubah T Partelli S Cives M Andreasi V Silvestris F Falconi M. Local treatment for focal progression in metastatic neuroendocrine tumors. Endocr Relat Cancer (2019) 26:405–9. 10.1530/ERC-18-0462 30668527

[15] Haraldsdottir S Hampel H Tomsic J Frankel WL Pearlman R de la Chapelle A. Colon and endometrial cancers with mismatch repair deficiency can arise from somatic, rather than germline, mutations. Gastroenterology (2014) 147(6):1308–16.e1. 10.1053/j.gastro.2014.08.041 PMC4294551 25194673

[16] Vanoli A Perfetti V Furlan D Neri G Viglio A Sessa F. Long Survival and Prolonged Remission after Surgery and Chemotherapy in a Metastatic Mismatch Repair Deficient Pancreatic Neuroendocrine Carcinoma with MLH1/PMS2 Immunodeficiency and Minimal Microsatellite Shift. Endocr Pathol (2020) 31:411–7. 10.1007/s12022-020-09622-5 32388775

[17] Sahnane N Furlan D Monti M Romualdi C Vanoli A Vicari E. Microsatellite unstable gastrointestinal neuroendocrine carcinomas: a new clinicopathologic entity. Endocr Relat Cancer (2015) 22(1):35–45. 10.1530/ERC-14-0410 25465415

[18] Serracant Barrera A Serra Pla S Blazquez Mana CM Salas RC Garcia Monforte N Bejarano Gonzalez N. Pancreatic non-functioning neuroendocrine tumor: a new entity genetically related to Lynch syndrome. J Gastrointest Oncol (2017) 8(5):E73–E9. 10.21037/jgo.2017.07.02 PMC5674252 29184699

[19] Qiu H Jin BM Wang ZF Xu B Zheng QF Zhang L. MEN1 deficiency leads to neuroendocrine differentiation of lung cancer and disrupts the DNA damage response. Nat Commun (2020) 11(1):1009. 10.1038/s41467-020-14614-4 32081882 PMC7035285

[20] Alexandrov LB Nik-Zainal S Wedge DC Aparicio SA Behjati S Biankin AV. Signatures of mutational processes in human cancer. Nature (2013) 500(7463):415–21. 10.1038/nature12477 PMC3776390 23945592

[21] Touat M Li YY Boynton AN Spurr LF Iorgulescu JB Bohrson CL. Mechanisms and therapeutic implications of hypermutation in gliomas. Nature (2020) 580(7804):517–23. 10.1038/s41586-020-2209-9 PMC8235024 32322066

[22] Pelosi G Bianchi F Dama E Simbolo M Mafficini A Sonzogni A. Most high-grade neuroendocrine tumours of the lung are likely to secondarily develop from pre-existing carcinoids: innovative findings skipping the current pathogenesis paradigm. Virchows Arch (2018) 472(4):567–77. 10.1007/s00428-018-2307-3 29388013

[23] Bahri H Laurence L Edeline J Leghzali H Devillers A Raoul JL. High prognostic value of 18F-FDG PET for metastatic gastroenteropancreatic neuroendocrine tumors: a long-term evaluation. J Nucl Med (2014) 55(11):1786–90. 10.2967/jnumed.114.144386 25286923

